# How Hypertension Affects Heart Metabolism

**DOI:** 10.3389/fphys.2019.00435

**Published:** 2019-04-16

**Authors:** Agnieszka Polak-Iwaniuk, Ewa Harasim-Symbor, Karolina Gołaszewska, Adrian Chabowski

**Affiliations:** ^1^Faculty of Health Sciences, Lomza State University of Applied Sciences, Łomża, Poland; ^2^Department of Physiology, Medical University of Białystok, Białystok, Poland

**Keywords:** DOCA-salt, FAT/CD36, heart metabolism, hypertension, SHR

## Abstract

Hypertension is one of the most frequently observed cardiovascular diseases, which precedes heart failure in 75% of its cases. It is well-established that hypertensive patients have whole body metabolic complications such as hyperlipidemia, hyperglycemia, decreased insulin sensitivity or diabetes mellitus. Since myocardial metabolism is strictly dependent on hormonal status as well as substrate milieu, the above mentioned disturbances may affect energy generation status in the heart. Interestingly, it was found that hypertension induces a shift in substrate preference toward increased glucose utilization in cardiac muscle, prior to structural changes development. The present work reports advances in the aspect of heart metabolism under high blood pressure conditions, including human and the most common animal models of hypertension.

## Introduction

Hypertension is one of the most widespread diseases of civilization, in which chronically elevated blood pressure (BP) induces ventricular hypertrophy and contractile dysfunction together with its clinical manifestations. Interestingly, in 2017 recommended threshold for hypertension diagnosis was set at 130/80 mm Hg (for details see Tables 1, 2) (Whelton et al., 2017) and consequently its prevalence increased in the general population. It was estimated that the number of patients suffering from this disorder is slowly reaching the number of normotensive persons (Whelton et al., 2017). Despite extensive knowledge about factors that promote hypertension (Table 3), determination of its etiology is a significant challenge for clinicians. In more than 90% of patients, the cause of hypertension is unknown (primary hypertension). Nevertheless, it is suspected that genetic predispositions and environmental factors play a crucial role in the development of the primary form of hypertension. In the remaining 10% of patients, high BP is the consequence of other medical conditions (Table 3). If a cause is correctly diagnosed, the secondary form of hypertension may be properly treated (Whelton et al., 2017). A meta-analysis of 61 prospective studies revealed a log-linear relationship between the increase in systolic blood pressure (SBP; from < 115 mm Hg to > 180 mm Hg) as well as diastolic blood pressure (DBP; from < 75 mm Hg to > 105 mm Hg) and increased risk of cardiovascular diseases (CVDs) (Lewington et al., 2002). Importantly, 20 mmHg elevated SBP aguments about 50% risk of heart failure (Haider et al., 2003). Since WHO designated CVDs as the predominant cause of death globally it is not surprising that hypertension is considered to be an important issue.

**Table 1:** Categories of blood pressure levels (mm Hg) – new classification recommended by the American College of Cardiology (ACC) and American Heart Association (AHA).

BP category	SBP (mmHg)		DBP (mmHg)
Normal	<120	and	<80
Elevated	120–129	and	<80
Hypertension			
Stage 1	130–139	or	80–89
Stage 2	≥140	or	≥90


*DBP, diastolic blood pressure; SBP, systolic blood pressure.*

**Table 2:** Categories of blood pressure levels (mm Hg).

BP category	SBP (mmHg)		DBP (mmHg)
Optimal	<120	and	<80
Normal	120–129	and/or	80–84
High normal	130–139	and/or	85–89
Hypertension			
Stage 1	140–159	and/or	90–99
Stage 2	160–179	and/or	100–109
Stage 3	≥180	and/or	≥110
Isolated systolic hypertension	≥140	and	<90


*DBP, diastolic blood pressure; SBP, systolic blood pressure.*

**Table 3:** Causes of hypertension.

Causes of hypertension (Whelton et al., 2017)		
**Genetic predisposition**	**Environmental risk factors**	**Diseases**
✓ Monogenic forms of hypertension:	✓ Overweight and obesity	✓ Renal parenchymal disease
– Glucocorticoid-remediable aldosteronism,	✓ Increased sodium intake	✓ Renovascular disease
– Liddle’s syndrome,	✓ Insufficient intake of potassium, calcium, magnesium, protein (especially from vegetables), fiber, and fish fats	✓ Primary aldosteronism
– Gordon’s syndrome,	✓ Poor diet	✓ Obstructive sleep apnea
✓ Polygenic disorders	✓ Lack or small physical activity	✓ Drug or alcohol induced pheochromocytoma/ paraganglioma
	✓ Alcohol consumption	✓ Cushing’s syndrome
		✓ Hypothyroidism
		✓ Hyperthyroidism
		✓ Aortic coarctation (undiagnosed or repaired)
		✓ Primary hyperparathyroidism
		✓ Congenital adrenal hyperplasia
		✓ Mineralocorticoid excess syndromes other than primary aldosteronism
		✓ Acromegaly


Moreover, it is suggested that approximately 45% of hypertensive patients have metabolic syndrome (Akholkar et al., 2015), indicating the role of hypertension in dysregulation of energy balance. Following this information, we decided to collect and summarize research in the field of metabolism in one of the most important organs affected by hypertensive state. This review describes myocardial energy substrate turnover during physiological state and its changes during increased BP conditions based on the results of tests carried out on humans and animals.

## Heart Metabolism

Long-chain fatty acids (LCFAs; 70–80%) and glucose (20–30%) are major energy substrates in the heart under normal, physiological conditions (Gimeno et al., 2003; Stanley et al., 2005). It is well-established that LCFAs are transported across the plasma membrane (PM) by protein-mediated transport (70%) or simple diffusion (30%) (Chabowski et al., 2008). To date, three main groups of LCFA transporters are identified: fatty acid translocase (FAT/CD36), PM-associated fatty acid binding protein (FABPpm) and fatty acid transport proteins 1–6 (FATP1-6; myocardial types 1, 4, and 6) (Bonen et al., 2002; Gimeno et al., 2003; Polak et al., 2016). Upon entering the cardiomyocytes, LCFAs are rapidly bound by the heart-type cytoplasmic fatty acid binding protein (H-FABP_C_) (Chabowski et al., 2008). Then, the enzyme fatty acyl-CoA synthetase (ACS) catalyzes the conversion of LCFAs into acyl-CoA (Faergeman and Knudsen, 1997). The activated LCFAs are bound by acyl-CoA binding proteins (ACBP) and subsequently they might be transported either into mitochondria for β-oxidation (70–80%) or esterified to distinct lipid pools (20–30%), mainly triacylglycerols (TAGs) (Luiken et al., 1997).

**FIGURE 1:**
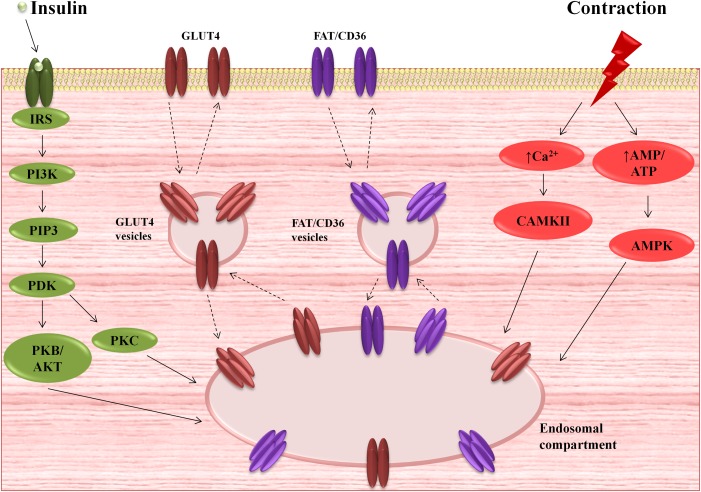
Myocardial activation of insulin and contractile signaling pathways and subsequent translocation of glucose transporter 4 (GLUT4) and fatty acid translocase (FAT/CD36) from the intracellular compartments to the plasma membrane (PM). AMPK, AMP-activated protein kinase; CAMKII, Ca^2+/^calmodulin-dependent kinase II; IRS, insulin-receptor substrate; PDK, phosphoinositide-dependent kinase; PI3K, phosphatidylinositol 3-kinase; PIP3, phosphatidylinositol 3-phosphates; PKB/AKT, serine/threonine protein kinase B; PKC, protein kinase C.

**FIGURE 2:**
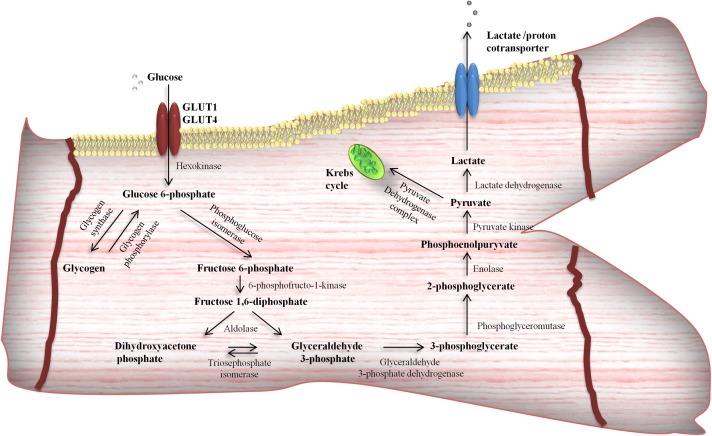
Glycolytic pathway in cardiomyocytes. Glucose after entering the cells via facilitated diffusion [glucose transporter 4 (GLUT4)] can undergo different intramyocardial metabolic pathways and become available for energy production or stored as a glycogen.

Due to its hydrophilic nature, glucose is unable to pass across the PM by simple, passive diffusion. In this regard, transmembrane uptake of glucose is regulated by special protein carriers (Becker et al., 2001; Szablewski, 2017). There are two families of transporters engaged in glucose uptake by cardiac cells – glucose transporters (GLUTs) and the sodium/glucose cotransporters (SGLTs). Currently, there is little information about SGLTs in the heart. It was found that SGLT1 is highly expressed in cardiac myocytes (Kashiwagi et al., 2015) and predominantly localized in the PM (Banerjee et al., 2009). Moreover, it is suggested that SGLT1 is sensitive to hormone stimuli (Banerjee et al., 2009). However, expression, cellular localization and the role of other SGLTs isoforms in cardiac myocytes is unknown (Szablewski, 2017). Recent data provide evidence for the existence of at least seven transporters belonging to GLUT family in the heart, i.e., GLUT1, GLUT3, GLUT4, GLUT8, GLUT10, GLUT 11, and GLUT 12 (Szablewski, 2017). The most important role in myocardial glucose flux is played by GLUT 1 and 4 (Zorzano et al., 1997; Szablewski, 2017). GLUT1, the isoform, which predominantly occurs in the fetal myocardium, is responsible for basal glucose transport and is mostly located at the PM (Zorzano et al., 1997; Becker et al., 2001). However, mature cardiomyocytes express GLUT4 to a greater extent than GLUT1 (Wang and Hu, 1991; Santalucía et al., 1992). In the resting state, most of the GLUT4 molecules reside in the intracellular, vesicular compartment (Becker et al., 2001). Its translocation to the cell surface is triggered primarily by insulin stimulation or cardiac contractile activity (Zorzano et al., 1997; Becker et al., 2001; Chabowski et al., 2006), which was shown in Figure 1. Activation of insulin pathway (e.g., post-prandial) leads to insulin-induced GLUT4 translocation and glucose influx into cardiomyocytes (Chang et al., 2004; Mora et al., 2004), promoting glycolysis (Depré et al., 1998), as well as myocardial glycogen synthesis (Goodwin et al., 1995). Interestingly, insulin also stimulates fatty acid transporters translocation (Luiken et al., 2002; Chabowski et al., 2004, 2005), mainly FAT/CD36 (Luiken et al., 2002), and concomitantly increases intramyocardial lipid deposition. On the other hand, metabolic demands of the working heart stimulate different cascades among which the major role is played by AMP-activated protein kinase (AMPK) signaling pathway (Luiken et al., 2004). Its activation is known to promote a variety metabolic actions, which in turn intensify glycolysis and fatty acid oxidation (Stanley et al., 2005). Glycolysis (Figure 2) is an oxygen-independent pathway. However, its products (pyruvate and NADH + H^+^) need oxygen for effective production of ATP (Stanley et al., 2005). When oxygen supply is poor, the pyruvate is converted into lactate in the process called anaerobic glycolysis, which becomes important in the preservation of myocardial energy-yielding process during ischemia or hypoxia incidents (Essop, 2007; Lopaschuk, 2017).

Nonetheless, the cardiac metabolic network is a flexible system that adapts to the current environment. Therefore, maintaining a high capacity for ATP production depends on numerous mechanisms. The first type of metabolic control is the classic allosteric regulation of the activity of key enzymes along the metabolic pathway. Allosteric control involves the binding of signaling molecules to specific regulatory sites, which induces conformational changes and alterations in the catalytic activity of enzymes (Kolwicz et al., 2013). For example, inactivation of pyruvate dehydrogenase (PDH) by pyruvate dehydrogenase kinase 4 (PDK4) is associated with a decrease in glucose catabolism and a corresponding rise in fatty acid oxidation in response to increased fatty acid availability during prolonged starvation (Wu et al., 2001; Sugden and Holness, 2003; Zhang et al., 2014). Additionally, a well-known regulator of fatty acid uptake and metabolism, malonyl-CoA, due to allosteric inhibition of carnitine palmitoyltransferase 1 (CPT1) regulates mitochondrial LCFA uptake and β-oxidation (Lopaschuk et al., 2010; Fillmore and Lopaschuk, 2014). A decreased malonyl-CoA level in pathological conditions, such as ischemia or diabetes, increases fatty acid oxidation rates at the expense of glucose metabolism, elevates the oxygen cost of contractility and decreases cardiac efficiency (Fillmore and Lopaschuk, 2014). The second mechanism, post-translational modifications (PTMs), changes the functional diversity of the proteome. The most common PTMs are covalent addition or removal of functional groups, the specific cleavage of precursor proteins and disulfide bonds formation (Lopaschuk, 2017). Accordingly, it was demonstrated that acetylation of mitochondrial enzymes, e.g., LCAD plays an important role in cardiac metabolism regulation. Studies conducted on mice lacking mitochondrial sirtuin 3 (SIRT3) have revealed that increased long-chain acyl coenzyme A dehydrogenase (LCAD) acetylation may be responsible for lower myocardial fatty acid oxidation (Hirschey et al., 2010; Chen et al., 2015). This information is especially important since the downregulation of SIRT3 protein has been implicated in heart failure in Dahl-salt sensitive and spontaneously hypertensive heart failure prone (SHHF) rats (Grillon et al., 2012). Another deacetylase namely SIRT 5, additionally exhibits desuccinylase and demalonylase activity. Additionally, absence of SIRT5 leads to suppression of PDH activity as well as glyceraldehyde 3-phosphate dehydrogenase (GAPDH) in SIRT5^-^/^-^ compared to wild type mice (Park et al., 2013; Nishida et al., 2015). Moreover, Hershberger et al. (2017) have demonstrated that SIRT5 is required for maintaining cardiac oxidative metabolism in response to cardiac pressure overload. Phosphorylation, as another way of enzyme modification, also plays an important role in the cardiac metabolism regulation. One of the previous studies showed that phosphorylation and subsequent inhibition of both acetyl-CoA carboxylase isoforms, ACC1/ACC2, suppressed acetyl-CoA to malonyl-CoA conversion and increased fatty acid uptake into mitochondria during reperfusion of ischemic rat hearts (Bairwa et al., 2016). Other PTM, such as *O*-GlcNAcylation may be associated with a significant increase in PM levels of FAT/CD36 and palmitate oxidation, which was observed in rats’ hearts subjected to a high glucosamine concentration. Similar changes reflecting increased fatty acid metabolism are observed in the early stages of diabetes (Laczy et al., 2011). The third mechanism is the regulation of gene transcription in which transduction of specific signal to the cell nucleus affects gene expression. This kind of control includes many of key transcriptional regulators of mitochondrial fuel metabolism and energy production pathways such as the peroxisome proliferator-activated receptors (PPARs) (Leone and Kelly, 2011; Lee et al., 2017), estrogen-related receptors (ERRs) (Wang et al., 2015) or nuclear respiratory factors 1 and 2 (NRF-1 and 2) (Dorn et al., 2015). Transcriptional control has an influence on the level of enzymes expression and acts on a longer time scale. Coordination of these various mechanisms ensures that the energy balance is maintained (Desvergne et al., 2006). The most known regulators of glucose and fatty acids metabolism are summarized in Table 4.

**Table 4:** Main regulators of glucose and fatty acids metabolism in the heart.

Action	Fatty acid oxidation	Glucose oxidation	Glycolysis
Stimulation	✓ Fatty acids (Fillmore et al., 2014)	✓ Insulin (Depré et al., 1998)	✓ Insulin, epinephrine (Depré et al., 1998)
	✓ Adiponectin (Onay-Besikci et al., 2004)	✓ Epinephrine (Karwi et al., 2018)	✓ AMP, ADP, Pi, NAD^+^ (Kolwicz, 2018)
	✓ Leptin (Hall et al., 2015)	✓ NAD^+^ (Kolwicz, 2018)	✓ Peroxisome proliferator-activated receptor gamma (Madrazo and Kelly, 2008)
	✓ Malonyl CoA decarboxylase (Dyck et al., 2004)		✓ AMP-activated protein kinase (Bairwa et al., 2016)
	✓ AMP-activated protein kinase (Lopaschuk et al., 2010)		✓ Hypoxia-inducible factor 1-alpha (Cerychova and Pavlinkova, 2018)
	✓ Peroxisome proliferator-activated receptors/peroxisome proliferator-activated receptor gamma coactivator 1α/estrogen-related receptors (Lee et al., 2017; Rowe et al., 2010; Huss et al., 2004)		
	✓ Forkhead box protein O1(Puthanveetil et al., 2013)		
Inhibition	✓ Glucose (Fillmore et al., 2014)	✓ Fatty Acids (Kodde et al., 2007)	✓ ATP, NADH (Kolwicz, 2018)
	✓ Lactate (Kolwicz, 2018)	✓ ATP, NADH, Acetyl CoA (Kolwicz, 2018)	✓ Glucose 6-phosphate (Depré et al., 1998)
	✓ Ketone bodies (Stanley et al., 2003)	✓ Pyruvate dehydrogenase kinase 4 (Fillmore et al., 2014)	✓ Citrate (Fillmore et al., 2014)
	✓ Malonyl-CoA (Fillmore et al., 2014)	✓ Peroxisome proliferator-activated receptor alpha (Lee et al., 2017)	
	✓ Acetyl CoA carboxylase 2 (Kolwicz et al., 2012)	✓ Forkhead box protein O1 (Gopal et al., 2017)	
	✓ Angiotensin II (Pellieux et al., 2009)	✓ Angotensin II (Mori et al., 2012)	


## Myocardial Metabolism in Hypertension

### Human Hypertension

An abnormal glucose metabolism, including insulin resistance, impaired glucose tolerance or diabetes mellitus as well as dyslipidemia are frequently diagnosed in patients with different types of hypertension (Onat et al., 2005; García-Puig et al., 2006; Chen et al., 2014). However, there are only a few reports investigating cardiac energy homeostasis in such patients. Importantly, de las Fuentes et al. (2003, 2006) demonstrated that patients with hypertension exhibited reduced myocardial fatty acid oxidation. Moreover, it was found this phenomenon preceded an increase in the left ventricle (LV) mass and might have been responsible for decreased myocardial efficiency (de las Fuentes et al., 2006). Presumably, the basis of this metabolic imbalance is complex and includes genomic alterations, e.g., variation in peroxisome proliferator-activated receptor alpha (PPARα) gene (Jamshidi et al., 2002). Taken together, these data support the hypothesis that disturbances in substrate metabolism may be related with morphological and functional abnormalities in the hypertensive heart.

To date, there are two contradictory reports concerning the influence of hypertension on glucose uptake in the human heart. Laine et al. (1999) found no differences in glucose uptake in hypertensive patients with or without LVH in comparison with the normotensive group. Whereas, Hamirani et al. (2016) observed higher myocardial glucose flux in the hypertensive group without LVH compared to the hypertensive patients with LVH and the normotensive group as well. However, myocardial glucose uptake in the hypertensive patients with hypertrophy was significantly reduced in comparison with the normotensive patients in this study (Hamirani et al., 2016). Discrepancies in glucose uptake rate may result from differences in age, body mass index as well as SBP value of patients in the above experiments. Nonetheless, it seems that an increase in glucose utilization results from pressure overload, but not hypertrophy, and occurs only in severe stages of hypertension (over 150 mm Hg SBP) or when the difference in SBP between the examined and control groups is sufficiently high (∼35 mm Hg) (Laine et al., 1999; Hamirani et al., 2016).

Interestingly, it has been shown that myocardial phosphocreatine/ATP ratio, a marker of an energetic state of the heart, was decreased in the hypertensive hypertrophied heart (Lamb et al., 1999). Probably, this resulted from reduced creatine kinase activity and lower total creatine content, which is frequently observed in the hypertrophied human heart (Ingwall et al., 1985). However, it has been proven that myocardial oxygen consumption per weight unit was elevated in the hypertensive patients without LVH, whereas, in those with LVH was normal (Laine et al., 1999). Nonetheless, oxygen consumption in the hypertensive hypertrophied heart at the same level as in the heart from healthy humans occurs to the detriment of cardiac efficiency. This in turn may predispose to heart failure development (Laine et al., 1999).

### Animal Models of Hypertension

#### Primary Hypertension

Spontaneously hypertensive rats (SHRs) introduced by Okamoto and Aoki (1963), are widely used in experimental research as genetic model of hypertension (Polak et al., 2018a). These rats develop hypertension without any physiological, pharmacological or surgical procedures.

Similarly to hypertensive patients, SHRs have been shown to be hyperglycemic and hyperinsulinemic (Gouveia et al., 2000; Daud et al., 2016). Moreover, it is suggested that these animals have reduced peripheral glucose utilization, which also makes them insulin resistant (Hulman et al., 1993; Gouveia et al., 2000; Zecchin et al., 2003). Bearing in mind that both glucose gradient across the PM and insulin action determine the rate of glucose influx and intracellular utilization, systemic abnormalities occurring in SHRs may directly affect myocardial metabolism (Zorzano et al., 1997). Intriguingly, it was demonstrated that the uptake of the glucose tracer 2-[^18^F]FDG was severalfold increased in SHR hearts (Hajri et al., 2001; Quintana-Villamandos et al., 2013; Wang et al., 2017; Li et al., 2018). Moreover, Hajri et al. (2001) investigation also found impaired myocardial uptake of fatty acid analog [^125^I]BMIPP. Other studies, which showed diminished palmitate oxidation and moderately elevated glucose oxidation in the perfused hearts, also revealed a shift toward glucose use instead of fatty acid in SHR strain (Christe and Rodgers, 1994, 1995). Furthermore, it was revealed that myocardial level of total carnitine was significantly reduced in SHR (Christe and Rodgers, 1995). Therefore, the above increase in glucose uptake reflects a compensatory response to defective myocardial fatty acid utilization, but not benefits of glucose and insulin abundance.

It should also be mentioned that the second genetic model of hypertension, namely Dahl-salt rats, presents similar to SHRs changes regarding substrate preference in myocardial metabolism (Yonekura et al., 1985; Kato et al., 2010). Why glucose replaces fatty acids, a main source of energy in the heart, is yet not known. It is well-known that about 10% more ATP is generated from glucose than fatty acid oxidation per mole of oxygen (Stanley and Chandler, 2002). Thus, the possible explanation of the switch in fuel type is adaptation to effective energy generation in adverse hemodynamic conditions, which was also reported in other models of the heart hypertrophy (Allard et al., 1994; Barger and Kelly, 1999).

Moreover, it is proposed that impairment in fatty acid metabolic rate intensifies with age in SHRs (Reutter et al., 2011). On the basis of the studies in which ^13^C-labeled energy substrates were used to determine SHR myocardial metabolism, it may be assumed that cardiac muscle of young (7-week-old) SHRs adopts more effectively to hypertension. It is by the fact that compensational increase in exogenous ^13^C-glucose use completely replaced reduced exogenous LCFA oxidation in the hearts of 7-week-old SHRs. Moreover, partitioning of exogenous ^13^C-glucose between glycolysis and oxidation through citric acid cycle was unchanged in the cardiomyocytes of 7-week-old SHRs compared to the normotensive rats (Lauzier et al., 2011). On the contrary, older rats (15-week-old) manifested a reduced contribution of exogenous ^13^C-labeled LCFA to myocardial energy production, which does not seem to be fully compensated by exogenous carbohydrates but endogenous energy sources. Surprisingly, the authors of these reports imply that these sources are either LCFA released from myocardial TAG stores or amino acids from enhanced proteolysis in the heart (Vincent et al., 2003; Labarthe et al., 2005). In addition, increased lactate release by the heart in 15-week-old SHRs (Vincent et al., 2003) also suggests decreasing capability of cardiac tissue in older animals to maintain its metabolic homeostasis. Despite these alterations, the SHRs display the same as in the normotensive control group myocardial oxygen consumption together with intracellular pH and, surprisingly, unaffected cardiac power and efficiency regardless of age (Vincent et al., 2003; Lauzier et al., 2011).

Interestingly, introduction of short-chain fatty acids (SCFAs) to SHRs’ diet prevented the heart hypertrophy development in spite of persistent hypertension (Hajri et al., 2001). A similar effect was observed in the *ex vivo* model of perfused SHR heart during medium-chain fatty acid (MCFAs) supplementation (Labarthe et al., 2005), which likewise SCFAs (and in contrast to LCFAs), do not require protein carriers (Schönfeld and Wojtczak, 2016). Two conclusions from these both experiments can be drawn. Firstly, abnormalities regarding myocardial fatty acid metabolism are involved in the development of hypertrophy in the SHR hearts. Secondly, it is possible that the hypertensive state affects fatty acid transport. Interestingly, Aitman et al. (1999) detected a defect in the gene of the major fatty acid transporter, FAT/CD36 in SHRs. This study also demonstrated that the encoded product of mutated FAT/CD36 gene was undetectable in PM of SHRs’ adipocytes, causing doubt whether SHRs are deprived of this protein transporter (Aitman et al., 1999). However, Gotoda et al. (1999) proved that FAT/CD36 mutation in the North American SHR strain used in Aitman et al. (1999) research is absent in the original SHR strains maintained in Japan. Additionally, Bonen et al. (2009) demonstrated that SHR strains express different FAT/CD36 mRNA. This phenomenon was not coupled with the absence of FAT/CD36 expression because its presence was observed in several tissues (including heart) of the North American SHR strain (Bonen et al., 2009). Therefore, the results obtained by Aitman et al. (1999) may be the consequence of inappropriate antibodies used instead of real absence of this protein (Bonen et al., 2009). Nevertheless, it should be pointed out that there was a decreased expression and plasmalemmal content of fatty acid transporters (total FAT/CD36: -26%, total FATP1: -35% and PM FAT/CD36: -36%) in the heart of 15-week-old SHRs (Bonen et al., 2009). Interestingly, the reduced FAT/CD36 protein level in PM and impaired fatty acid utilization occurs also in 7-week-old SHRs, prior to developing LVH (Lauzier et al., 2011). This may imply that the onset of myocardial metabolic disturbances appears during the early stages of hypertension and probably precedes anatomical and functional changes in the heart. Moreover, data indicate susceptibility of FAT/CD36 in the SHR hearts to defective PTMs, specifically to N-glycosylation (Lauzier et al., 2011). The authors of this report pointed out that the above change could be responsible for the lowered plasmalemmal FAT/CD36 content as well as loss of its function, which was followed by reduced capacity for fatty acid transport (Lauzier et al., 2011). Moreover, the studies conducted on SHR-4, a congenic SHR strain with inserted functional FAT/CD36 gene segment (from the Brown Norway strain), revealed that FAT/CD36 mutation in SHR may have influence on LVH development (Klevstig et al., 2011). Interestingly, the alterations in FAT/CD36 level or its function were linked with diminished fatty acid metabolism, elevated cardiovascular risk and heart diseases also in humans (Febbraio and Silverstein, 2007; Kim and Dyck, 2016).

Additionally, there is evidence that hypertension can affect intracellular signaling pathways in the heart which are crucial for maintaining energy substrate transport and myocardial energetic status. Accordingly, it was noted that spontaneous mutation in Pflz gene, occurring in SHR, probably is associated with disturbances in myocardial PPARα and insulin signaling at the genome level (Liška et al., 2017). Furthermore, it was demonstrated that PKCε expression as well as its activation were decreased in the SHR’s cardiac muscle which carries a non-functional variant of the FAT/CD36 (Klevstig et al., 2011). The authors of this report do not exclude the possibility that this phenomenon may be the basis of impaired LCFA transport in the cardiomyocytes (Klevstig et al., 2011). What is more, Dolinsky et al. (2009) showed that phosphorylation of liver kinase B (LKB1) and its direct substrate, AMPK, was significantly reduced in the 15-week (but not in 7-week) SHR hearts compared to the hearts of normotensive rats. Interestingly, this inhibition of the LKB1-AMPK signaling pathway was related to cardiac hypertrophy, but not intracellular energy status because AMP/ATP ratio was unchanged (Dolinsky et al., 2009). Similarly, Christe and Rodgers (1995) also observed that the levels of myocardial high-energy phosphates (ADP, AMP, and ATP) were unaffected in the SHRs, which indicates that the total energy production in myocardium is stable despite of hypertension.

Nonetheless, it should be mentioned that in SHR hearts disorders regarding intracellular energy substrates stores can appear. For example, there is proof for DAG to TAG conversion inhibition, since [^125^I]BMIPP was incorporated more intensively into DAG than TAG myocardial fraction (Hajri et al., 2001). On the other hand, study conducted by LaPier and Rodnick (2000) revealed that 22- and 14-week-old SHRs exhibited a significantly elevated myocardial TAG content compared to 6-week-old individuals, which proved that TAG accumulation may intensifies with age. The same study also showed that hypertension decreased myocardial glycogen content in all the examined age-matched groups (LaPier and Rodnick, 2000). Moreover, LaPier and Rodnick (2000) demonstrated that hypertensive rats had an elevated plasma glucose availability as well as unchanged myocardial GLUT1 and GLUT4 expression, indicating an unaffected capacity for glucose transport into cardiomyocytes in hypertension. Thus, glycogen depletion probably resulted from its increased use due to pressure overload heart as well as confirmed a shift in substrate preference (LaPier and Rodnick, 2000). It is interesting to note that LaPier and Rodnick (2000) studies have been carried out on female SHRs and observed changes are consistent with those in male SHR hearts, suggesting lack of gender differences in cardiac metabolism in hypertension.

#### Secondary Hypertension

##### Renal models

Renal hypertension is induced in animals using surgical procedures, which cause renal parenchyma or renal arteries damages (Polak et al., 2018a). Among all renal hypertension types, the most popular are models introduced by Goldblatt, in which elevation of BP is performed by a constriction of one or both renal arteries using a small clamp. For example, in one-kidney one-clip model (1K1C; one renal artery is constricted and concomitantly the contralateral kidney is removed) mild hypertension causes functional changes without concomitant hypertrophy of the LV, as seen in other animal models (Taegtmeyer and Overturf, 1988). These functional alterations are accompanied by myocardial metabolic changes favoring glucose as a fuel (enhanced glucose utilization and intensified activity of key glycolytic enzymes – hexokinase, phosphofructokinase, and phosphorylase) and decreased ketone bodies metabolism (Taegtmeyer and Overturf, 1988). In this case increased glucose utilization was not coupled with changes in lactate production indicating an increased rate of its oxidation but not the contribution in anaerobic metabolism. However, unchanged activities of two main enzymes of citric acid cycle [citrate synthase (CS) and 2-oxoglutarate dehydrogenase (Taegtmeyer and Overturf, 1988)] reflects similar to observed in SHR an unaltered rate of total energy production in spite of a shift in the myocardial metabolism toward glucose in the 1K1C model of hypertension.

Intriguingly, there are some differences amongst Goldblatt’s hypertension models. The study concerning enzyme activities of the energy-supplying metabolism revealed that myocardial CS activity in two-kidney one-clip rats (2K1C; one renal artery is constricted and the contralateral kidney is left intact) was reduced (Koehler and Medugorac, 1985), which is contradictory to the findings in the 1K1C model (Taegtmeyer and Overturf, 1988). Moreover, the study conducted by Smith et al. (1990) revealed nearly twofold lactate dehydrogenase (LDH) activity elevation after 6 weeks from the surgical procedure in the LV as well as right ventricle (RV) in the 2K1C model. The report of Koehler and Medugorac (1985) also noted that LDH activity in the LV in the initial phase of hypertension induction (4–6 weeks post-operatively) was significantly higher than in the LV of the normotensive group, although the increase was 6%. Nevertheless, this phenomenon was not observed in the further stages of the experiment (8–12 weeks post-operatively), and therefore Koehler and Medugorac (1985) proposed that this initially elevated LDH activity was an artifact of a surgical operation. It is interesting that the more pronounced changes were detected in Smith’s experiment (Smith et al., 1990), which was performed on 4-weeks rats [vs. 8-weeks rats in Koehler investigation (Koehler and Medugorac, 1985)]. A possible explanation may be that induction of hypertension in younger subjects caused immediate return to “fetal” metabolism, in which anaerobic conditions favor use of glucose and lactate (collectively referred to as carbohydrates) as the main energy sources (Taegtmeyer et al., 2010). This suggestion may be confirmed by a decreased activity of adult-type creatine kinase isoenzyme (CK-MM) and concomitantly elevated activity of the fetal-type CK isoenzymes (CK-MB+BB) in the heart of these animals (Smith et al., 1990). Moreover, a decreased total myocardial CK activity in 2K1C model probably indicates similar to hypertensive patients, impaired energy transfer to sarcomeres (Smith et al., 1990). However, a reduced activity of β-hydroxy-acyl-CoA dehydrogenase (β-HADH), which catalyzes the third step of β-oxidation, and increased hexokinase activity (Taegtmeyer and Overturf, 1988), are in line with the findings showing a shift in myocardial metabolism toward increased glucose utilization due to hypertension (Christe and Rodgers, 1994; Hajri et al., 2001).

##### Endocrinal models

Administration of mineralocorticoids or their synthetic derivatives, such as deoxycorticosterone acetate (DOCA) combined with sodium chloride, in unilateral nephrectomised rats produces volume overload hypertension (Lee et al., 2015). This model represents the form of endocrine hypertension observed in humans with primary aldosteronism. Interestingly, the DOCA-salt rats exhibited reduced insulin concentration in the blood (Dai and McNeill, 1992; Dai et al., 1992; Ndisang and Jadhav, 2010), which probably resulted from destruction of β-cells of pancreatic islets (Ndisang and Jadhav, 2010), similarly to humans with primary aldosteronism (Corry and Tuck, 2003). However, the observed decreased insulin concentration in this model did not affect glycemia (Dai and McNeill, 1992; Dai et al., 1992; Ndisang and Jadhav, 2010). Recent data revealed that DOCA-salt rats exhibit increased tissue insulin sensitivity (Polak et al., 2018b). It is interesting that DOCA-salt rats present similar to other animal models of hypertension a shift toward enhanced carbohydrates utilization in the heart, especially deriving from intramyocardial glycogen (Coprean et al., 1993). Presumably, this process occurred under aerobic conditions since elevated pyruvate dehydrogenase (PDH) expression was observed in the heart of DOCA-salt rats (Polak et al., 2018b). Moreover, recent studies noted unchanged myocardial expression and subcellular redistribution of two main glucose transporters, GLUT1 and GLUT4 in the DOCA-salt rats (Atkins et al., 2001; Polak et al., 2018b). Because glucose transport is regulated by protein carriers (Zorzano et al., 1997), this data imply the notion that glucose influx to cardiomyocytes in this model probably is unaffected by hypertension. In line with previous findings in SHR model, palmitate oxidation was decreased in the heart of DOCA-salt rats in which hypertension was induced already at 6–7 week old (Polak et al., 2017). On the contrary to SHRs, the fatty acid uptake seems to be unaffected, since myocardial palmitate uptake, as well as expression and subcellular redistribution of the primary fatty acid transporters (FAT/CD36, FATP1, FATP4, and FABPpm), were unchanged in these rats in comparison with the normotensive rats (Polak et al., 2017). Moreover, there were no changes in the activity of major proteins in signaling pathways stimulating transmembrane transport of energy substrates, such as AMPK, serine/threonine protein kinase B (AKT) and mitogen-activated protein kinase (MAPK) (Polak et al., 2018b). Nonetheless, there is some evidence that hypertension promotes metabolic changes in an age-dependent manner, also in this model of hypertension. In particular, this was indicated by an increased level of plasma lipids [cholesterol, free fatty acids (FFA), phospholipids (PL), and TAG] and their enhanced accumulation in the heart of DOCA-salt rats in which induction of hypertension was initiated at 11–13 week old (Prahalathan et al., 2012) [vs. unaffected plasma and myocardial lipids content in rats in which hypertension was induced at 6–7 week old (Polak et al., 2017)].

Collectively, DOCA-salt rats have primarily increased carbohydrates metabolism in the heart probably due to enhanced endogenous myocardial sources (glycogen) utilization. On the other hand, disturbances in energy generation from LCFAs are mainly related to their impaired oxidation, but not to transport into cardiomyocytes.

## Cardiac Metabolism in the Failing Heart

In hypertensive patients, pressure overload leads to cardiac remodeling including eccentric LV hypertrophy. When this state is sustained, there develops a diastolic dysfunction, decompensation of remodeled LV and finally hypertensive heart failure (Messerli et al., 2017). A growing body of evidence indicates that metabolism in the failing heart is impaired (Doehner et al., 2014). One of the most important alterations is 30–40% decreased ATP production, which results from disturbances in a mitochondrial function and decreased oxidative capacity (Rosca and Hoppel, 2013). Moreover, it is proposed that the failing heart switches from fatty acids oxidation to glucose metabolism to generate energy (Riehle et al., 2011; Kolwicz et al., 2012). However, this switch reflects an increase in glucose uptake and glycolysis instead of mitochondrial oxidative metabolism (Allard et al., 1994; Stanley et al., 2005; Lopaschuk et al., 2010). Uncoupling of glucose metabolism increases lactate and protons production (Folmes et al., 2006; Fillmore et al., 2018; Saleme and Sutendra, 2018). This, in turn, may reduce contractility of the heart, e.g., by decreasing troponin I sensitivity to calcium and inhibiting the slow calcium current (Morimoto and Goto, 2000). Furthermore, the utilization of ATP to eliminate protons and preserve calcium and sodium homeostasis worsens cardiac efficiency and function (Lopaschuk et al., 2010). Additionally, it is suspected that uncoupling glycolysis from glucose oxidation supports development of heart hypertrophy and its progression (Fillmore et al., 2018).

## Conclusion

**FIGURE 3:**
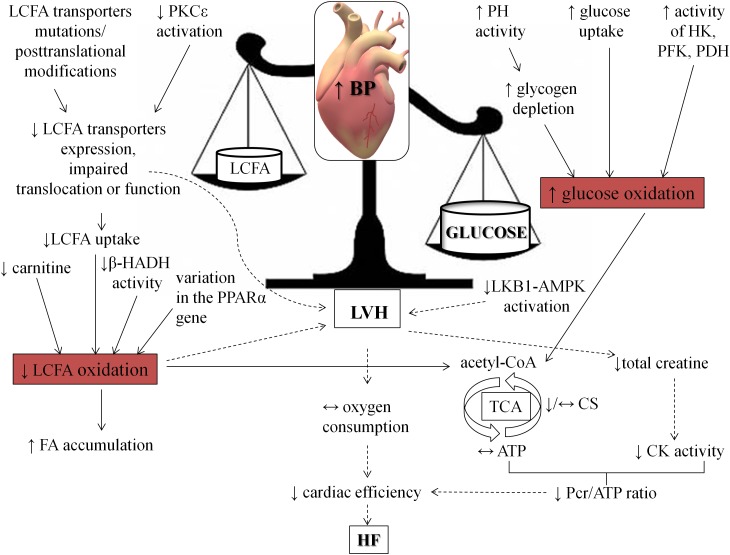
Consequences of hypertension on heart metabolism and function. The figure shows how hypertension shifts fuel preference from fatty acids toward carbohydrates. AMPK, AMP kinase; β-HADH, β-hydroxy-acyl-CoA dehydrogenase; CK, creatine kinase; CS, citrate synthase; HF, heart failure; HK, hexokinase; LKB1, liver kinase B; LVH, left ventricle hypertrophy; Pcr, phosphocreatine; PDH, pyruvate dehydrogenase; PFK, phosphofructokinase; PH, phosphorylase; PKCε, protein kinase C ε; PPARα, peroxisome proliferator-activated receptor alpha.

Myocardial energy metabolism is a complex process, which consists of a network of biochemical reactions and pathways. The initial stage is energy substrate utilization due to its uptake and metabolism as well as following participation of intermediates in TCA cycle. Then mitochondrial oxidative phosphorylation enables generation of ATP molecules, and finally, the creatine kinase system transports ATP from mitochondria to the sarcomeres. In the light of the foregoing evidence, metabolism of the heart subjected to pressure overload may be disturbed in all of this areas. In spite of differences among humans and animals, one of the main disorders during hypertension includes a shift from fatty acids toward enhanced carbohydrates utilization in the heart. What is interesting, these abnormalities are connected with impaired myocardial function (Figure 3). Therefore, implementation of strategies aimed at improving cardiac metabolism in BP lowering therapy seems to be extremely important (Swislocki et al., 1999; Prahalathan et al., 2012; Wang et al., 2017; Polak et al., 2018b; Harasim-Symbor et al., 2019).

## Author Contributions

AP-I participated in the design of the work, drafted the manuscript, prepared figures and tables and approved final version submitted. EH-S and KG helped to draft the manuscript and approved final version submitted. AC participated in the design of the study, revised manuscript and approved final version submitted. All authors agreed to be accountable for all aspects of the work.

## Conflict of Interest Statement

The authors declare that the research was conducted in the absence of any commercial or financial relationships that could be construed as a potential conflict of interest.
